# Conjugated linoleic acid modifies transcriptional cytokine profile and induces early specific secretory IgA response in *Giardia lamblia* infected mice

**DOI:** 10.22038/IJBMS.2022.65796.14471

**Published:** 2022-12

**Authors:** Itzel Reyes-Duarte, Alexel J. Burgara-Estrella, Lorena Bustamante-Córdova, Silvia Y. Moya-Camarena, Héctor Parra-Sánchez, Osiris Álvarez-Bajo, Guillermo López-Cervantes, Maricela Montalvo-Corral

**Affiliations:** 1 Departamento de Nutrición y Metabolismo, Centro de Investigación en Alimentación y Desarrollo, A.C, Hermosillo, México; 2 Departamento de Investigación en Física, Universidad de Sonora, Hermosillo, México; 3 CONACYT-Departamento de Investigación en Física, Universidad de Sonora, Hermosillo, México; 4 Departamento de Ciencias de la Salud, Universidad de Sonora, Hermosillo, México

**Keywords:** Conjugated linoleic acid, * Giardia lamblia*, IL-10, Secretory IgA, TGF-β

## Abstract

**Objective(s):**

Adaptive immunity is crucial in controlling *Giardia lamblia* infection in the intestinal mucosa, and some dietary lipids may improve mucosal immune function. The aim of this study was to evaluate conjugated linoleic acid (CLA) on the Th17/Treg response and secretory IgA production in a model of giardiasis infection.

**Materials and Methods:**

C3H/HeN male mice were infected with 5×10^6^ *G.* *lamblia* trophozoites (GS/M-83-H7, ATCC collection). Mice were assigned randomly to experimental and control groups. CLA was administered to the experimental group and phosphate-buffered saline (PBS) was given to the control group. Parasite load kinetics was determined. Enzyme-linked immunosorbent assay (ELISA) was performed to evaluate IgA and cytokines. Nuclear transcription factors and cytokines were measured by RT-qPCR, and histology of small bowel cells was evaluated.

**Results:**

CLA administration reduced the parasite load (*P<*0.05) and increased early *Giardia*-specific secretory IgA production. CLA also increased the expression of interleukin-10, transforming growth factor (TGF)-β, and inducible nitric oxide synthase (iNOS) (*P<*0.05), while infection elevated the expression of Foxp3, with a peak at 40 days post-infection (*P<*0.05). There were no pathological changes in the colonic mucosa due to infection or treatment. Thus, CLA stimulated mucosal immunity and enhanced the humoral response against *G. lamblia*, not only for early infection control but also to promote regulatory cytokine production at 40 dpi, restoring the intestinal balance after parasite elimination.

**Conclusion:**

Our findings reveal novel anti-parasitic effects through the immune-modulatory activity of CLA against the intestinal parasite *G. lamblia*.

## Introduction

*Giardia lamblia* (syn. *Giardia intestinalis*, *Giardia duodenalis*) is a non-invasive zoonotic flagellated protist that inhabits the proximal region in the small intestine of mammalian hosts (1). This protozoan is the etiologic agent of giardiasis, one of the more prevalent parasitic diseases worldwide, with 280 million people infected per year (2). *Giardia *spp. is a luminal pathogen causing symptomatic and asymptomatic infection for 1 or 2 weeks, cleared mainly by efficient immune responses. However, chronic conditions lasting months in vulnerable populations such as children, the elderly, and even immunocompetent people may occur (3). The mechanisms of parasite control have been studied over the last 20 years involving innate and adaptive responses (4, 5), while a humoral immune response is crucial for enteric *G. lamblia* infection resolution (6, 7). In particular, mucosal secretory IgA (sIgA) production in the intestine is an essential branch of defense against vegetative and infective forms of *Giardia* spp. (8, 9).

It is known that modifying the innate immune response leads to a change in the differentiation of virgin T cells into effector Th1, Th2, Th17, or regulatory T (Treg) cells, involved in the early elimination of infection (10). In the intestinal mucosal, Th17 cells and Tregs have a crucial role in the extracellular response against protozoan parasites, and their interactions differentiate between damage and tissue protection. 

Despite the knowledge concerning the immune system responses against *Giardia *and its importance in public health, no vaccine is available for humans; the current pharmacological treatment is effective, but there are non-desirable secondary effects, and the susceptible population still suffers the consequences of infection (11, 12). Immuno-nutrition provides an alternative approach to improve host defenses against infection, inflammation, and injury or damage. Nutrients have shown benefits beyond their nutritional and metabolic activity on immune system cells (13). Conjugated linoleic acid (CLA), present in red meat and dairy products, is a lipid family of geometric and positional isomers derived from linoleic acid (14). Extensive research has been performed on biological CLA activity showing promising findings that include unique properties to modulate immunomodulatory effects on the mucosa, such as its ability to enhance the humoral immune response in animal models and humans (13, 15-19). This study aimed to evaluate the activity of CLA through modulation of mucosal adaptive responses based on sIgA production as well as dynamic changes in the cytokine profile and characteristic transcription factors of Th17 cells in a mouse model of giardiasis.

## Materials and Methods

***Animals, diet, and treatments***

C3H/HeN male mice were purchased from the supplier (Harlan, Wilmington, MA, USA), and housed in the animal facility of the Research Center for Food and Development, at 21±2 ^°^C, 60% relative humidity, a 12 hr photoperiod (lights on from 07:00-19.00 hr), and provided with sterilized water and food *ad libitum. *The commercial rodent diet supported animal feeding for all mouse groups (Teklad Global Diet, Harlan, IN, USA). We conducted two bioassays. In the first one, 48 male mice (6-8 weeks old) were randomly assigned to two experimental groups following a 2×6 factorial design to monitor parasite load kinetics and a 2×4 design to evaluate the humoral response. CLA was administered to the experimental group (n=24) and phosphate-buffered saline (PBS) was given to the control group (n=24). Supplementation by oral gavage with 50 mg/day CLARINOL80® CLA free fatty acids 1:1 mixture of 40% *cis*-9,*trans*-11 CLA and 40% *trans*-10,*cis*-12 CLA isomers (provided by Stepan Nutrition Specialty Products LLC, NJ) was administrated for a total of 9 days, 3 days before and 6 days after infection. These conditions were repeated in a second experiment to assess cytokine production, nuclear transcription factor data, and histology of small bowel cells, in a 2×4 factorial design. The protocols were carried out in compliance with the institutional guidelines for the care and use of laboratory animals by the Bioethical Committee at Centro de Investigación en Alimentación y Desarrollo, A.C. (CE/005/2013).

***Experimental infection***

*G. lamblia* trophozoites (GS/M-83-h7, ATCC collection) were cultured in TYI-S-33 medium (20). The culture was harvested after 48-72 hr, during the log phase. Trophozoites were detached from polycarbonate tubes, chilled on ice, and collected by centrifugation at 400 *g* for 8 min (Beckman Coulter, USA). The cells were counted in a hemocytometer Neubauer chamber and adjusted to the desired cellular density. On the third day of administration of CLA or PBS, 20 mice per group were infected with 5×10^6^ *G. lamblia* trophozoites in 0.2 ml of PBS by oral gavage with a plastic feeding syringe to prevent tissue damage (Instech Solomon Laboratories, Plymouth Meeting, PA, USA). The syringe needle was previously immersed in 1% sucrose to promote more cooperative behavior in mice, which facilitated the experimental procedure and diminished animal stress. The remaining eight mice were inoculated with sterile PBS (0.2 ml) and considered the non-infected group at time 0. In addition, animals were fasted overnight to be more prone to swallow liquid content. Four mice in each group were euthanized by cervical dislocation at 0, 6, 14, 21, 28, and 40 days post-infection (dpi) to collect biological samples for antibody and trophozoite counts, and at 0, 6, 14, 21, and 40 dpi to obtain intestinal cells for population evaluation.

***Parasitic load***

The small intestine of each mouse (n=4 per group and per time) was removed aseptically to monitor the kinetics of infection (at 6, 14, 21, 28, and 40 dpi). Duodenal tissue (10 cm) was cut, opened longitudinally, and placed in PBS for 1.5 hr at 4 ^°^C in semi-horizontal agitation to detach *Giardia* trophozoites from the intestinal wall. Trophozoites were counted using a hemocytometer to calculate the parasitic load. 

***Fecal extract for immunoglobulin determination***

Fecal extract was obtained by following a previously reported technique (21), with minor modifications. In brief, individual mice were allocated in polystyrene cages for 1 hr to recover feces. Seven pieces of fecal content were obtained from each mouse at 0, 6, 14, 21, 28, and 40 dpi. Pellets in 0.5 ml of PBS 0.1% albumin (PBA), with 100 U/ml penicillin, 100 μg/ml streptomycin, and 0.1 ml of protease inhibitor cocktail (SIGMA, SL, USA) were incubated for 1 hr at 4 ^°^C. Vials with fecal homogenate were vortexed and centrifuged at 10,000 g for 10 min at 4 ^°^C to remove debris. Fecal extract supernatants were collected and stored at -80 ^°^C. The protein concentration of the fecal preparation was determined by using a protein assay kit (BCA, IL, USA).

***sIgA production***

*G. lamblia* anti-IgA antibodies were assessed in fecal extracts using an enzyme-linked immunosorbent assay (ELISA). In brief, 96 Maxisorp plates (Thermo Fisher Scientific, UK) were coated with 50 μg/ml of *Giardia* soluble extract in 100 μl of carbonate buffer per well and incubated overnight at 4 ^°^C. Culture plates were washed three times with PBS-0.05% Tween 20 (PBS-T), blocked with 5% fish gelatin (SIGMA, SL, USA) for 2 hr at room temperature, and washed three times with PBS-T. Later, 100 μl of 1:5 fecal extract homogenates were added and incubated for 1 hr at 37 ^°^C. Culture plates were washed four times and incubated with 1:500 biotinylated anti-mouse IgA for 1 hr (BioLegend, UK). Plates were then washed four times and incubated with 100 μl of horseradish peroxidase (HRP)-conjugated avidin 1:2000 for 1 hr. Then, 100 μl of 3,3′,5,5′-tetramethylbenzidine (TMB) was used as substrate, and color development was stopped with 50 μl of 1 M H_2_SO_4_. Optical density was measured in an ELISA microplate reader (Bio-Rad, Hercules, CA, USA) at 450 nm. Negative controls (t0, non-infected mice fecal extracts) and blanks were run in each assay to assess background reactivity, and optical density negative values plus a twofold standard deviation were considered to establish the cut-off limit of detection of *Giardia*-specific sIgA.

***G. lamblia soluble extract preparation***

Based on a previous study (7), water-soluble protein extract was obtained with a few modifications. In brief, 5×10^7^ trophozoites were washed three times with sterile PBS. Later, they were suspended in 0.5 ml PBS with 0.1 ml of protease inhibitor cocktail containing 4-(2-aminoethyl)benzenesulfonyl fluoride (AEBSF), pepstatin A E-64, bestatin, and sodium EDTA (Sigma, SL, MO, USA). Samples were frozen in liquid nitrogen and thawed at room temperature for three cycles, and then ultra-sonicated in an ice bath for 7 cycles of 10 pulses (Branson Sonifier 450, CT, USA). Cell debris was removed by centrifugation at 10,000 g for 30 min (Centrifuge 541r, Eppendorf, USA). The extract was filtered with a 0.22 µm membrane (Millipore, USA). The protein concentration of the soluble antigen preparation was determined using a protein assay kit (BCA, IL, USA). 

***Collection of intestinal tissue and isolation of cells***

Animals were euthanized by cervical dislocation. Intestinal cells were isolated using a previously reported protocol (22) with some modifications (23). In brief, the small intestine was surgically removed in aseptic conditions, sectioned, opened longitudinally, and washed with PBS to detach all fecal material. The tissue was cut into 5 cm pieces and incubated for 30 min at room temperature with Hank’s solution with 0.5 mM EDTA, 100 IU penicillin, and 1 mg/ml amphotericin B. Later, the tissue was washed with Hank’s solution with 20 mM HEPES, antibiotics, and 2% fetal bovine serum (FBS, Gibco, NY,USA). Tissue was cut into 0.5 cm pieces and transferred into a 25 ml flask with RPMI-1640 enzymatic solution [2 mM L-glutamine, 1 mM sodium pyruvate, 0.05 mM 2-mercaptoethanol, 100 IU penicillin (all from Sigma-Aldrich, USA), 1 mg/ml amphotericin B solution, 10% FB (Gibco, NY, USA), 4.25 µg/ml DNAse I, and 25 µg/ml collagenase]. The tissue was incubated for 2 hr at 37 ^°^C and 150 rpm in an orbital shaker (Incu-Shaker, Benchmark, NJ, USA). After incubation, the samples were macerated and filtered in a strain mesh of 100 µm. Supernatant and cells were collected in a 50 ml tube with cold RPMI-1640 (Gibco, MD, USA) complete medium. Later, the cell suspension was centrifuged at 400 *g* for 7 min at 4 ^°^C (Allegra 6R Centrifuge, Beckman Coulter, USA) and washed with cold RPMI-1640. Cells were counted in a hemocytometer, assessing viability by 0.4% trypan blue dye exclusion. 

***Isolating and culturing spleen cells***

The spleen was removed aseptically and collected in a 5 ml tube (BD, USA) with 1 ml of cold RPMI-1640 for its transportation. Subsequently, the spleen was macerated and filtered with fabric mesh. The cell suspension was collected and incubated with 5 ml of lysis solution (ammonium chloride, potassium bicarbonate, and EDTA) for 5 min to remove red blood cells. RPMI medium supplemented with 10% FBS was added to stop the lysis reaction. Cells were centrifuged at 329 g for 8 min at 4 ^°^C and then washed again. Splenocytes were divided into three groups of 2×10^6^ cells per well and incubated for 20 hr at 37 ^°^C in 5% CO_2_. One group did not receive any treatment, one group was stimulated with 20 µg/ml phorbol-myristate-acetate and ionomycin (PMA+I) (Life Technologies, MD, USA), and one group received 50 µg/ml soluble protein extract of *G. lamblia*. 

***Cytokine production measured by ELISA***

Supernatant collected from stimulated splenocytes was collected and cytokine production was measured using ELISA Ready-Set-Go! Kits (eBioscience, CA, USA) following the manufacturer’s instructions. Maxisorp® 96 well microplates were coated overnight with capture antibody and incubated at 4 ^°^C. Plates were blocked with ELISA diluent for 1 hr and then washed. Standards and samples were prepared and added to corresponding wells and incubated for 2 hr at room temperature. Secondary antibody was added and incubated for 1 hr. Later, avidin-HRP was added and incubated for 30 min. After washing the plate, 3,3′,5,5′-Tetramethylbenzidine (TMB) solution was added and incubated for 15 min. The reaction was stopped with 2% sulfuric acid (H_2_SO_4_). The final concentration of cytokines in untreated cells was subtracted from cells stimulated with soluble extract of *G. lamblia *or with PMA+I (as a positive stimulation control).

***RNA isolation from intestinal cells***

Total RNA was extracted from 3×10^6^ intestinal cells previously snap-frozen at -80 ^°^C with TRIzol (Invitrogen) following the manufacturer’s suggestions. Then, 500 µl of 99.5% chloroform was added to the samples, and they were vortexed and centrifuged at 17,950 g for 10 min at 4 ^°^C. The upper phase was separated and mixed with 200 µl of isopropanol. After 5 min, total RNA was recovered by centrifugation, washed with 1 ml of ethanol, centrifuged, and resuspended in 40 µl of nuclease-free water. The concentrations and 260/280 ratio (a measure of purity) were measured in a NanoDrop ND-1000 spectrophotometer (NanoDrop Technologies, USA).

***Gene expression analysis by reverse transcription–quantitative real-time polymerase chain reaction (RT-qPCR)***

The transcriptional profile of Th17 cells and Tregs (Table 1) was performed by reverse transcription with commercial system Brilliant II SYBR® Green QRT-PCR Master Mix (Applied Biosystems, USA). One hundred nanograms of total RNA and 200 nM of initial oligonucleotides sense and antisense in a reaction mixture of 25 µl (Table 1) were used. Thermal cycling was carried out as follows: 30 min at 50 ^°^C; 10 min at 95 ^°^C; 40 cycles of 95 ^°^C for 30 sec and 60 ^°^C for 30 sec. β-Actin was used for data normalization. Relative quantification for fold increase was calculated with 2^△△^^Ct ^using the previously reported formula (24). 

***Colon histology***

Samples of the ascending colon were taken at day 0 (pre-infection) and at 40 dpi (post-parasite elimination) to evaluate the effect of CLA on the colonic mucosa after expulsion of *G. lamblia*. A small section of the ascending colon was cut and fixed in 10% formaldehyde and embedded in paraffin. Three micrometer tissue sections were cut and stained with hematoxylin and eosin, following the standard technique.

***Statistical analysis***

A 2×4 factorial design for mouse weight, IgA response, and cytokines; a 2×3 factorial design for RT-qPCR (to 0, 6, 14, and 40 dpi); and a 2×6 factorial design for trophozoite count of CLA and control groups were tested for the main effects of supplementation and time, and their interaction. Statistical analysis was performed in R version 4.0.5 (www.R-project.org) using the rcompanion package (Mangiafico, S.S. 2016). The Scheirer-Ray-Hare test was performed to identify differences between factors and interactions. A post hoc Dunn’s test with the Benjamini–Hochberg method was performed to determine the significant factors (*P≤* 0.05). The Spearman test was used to evaluate correlations between antibody levels and parasite loads. *P<*0.05 was considered significant.

## Results

***Parasitic load in the small intestine***

**Figure 1:**
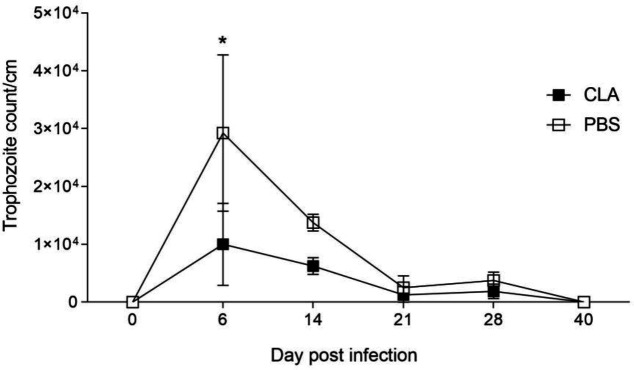
*Giardia lamblia* trophozoite kinetics in male C3H/HeN mice

We assessed the experimental infection by monitoring the weight of mice and the parasite load on different experimental days. The weights were homogeneous at the beginning of the assay with no differences before infection in the CLA and PBS groups (26.2 g±1.5 g *vs.* 24.5 g±0.5 g, respectively). After infection, the parasite did not affect body weight of the mice at 40 dpi (29.6 g±1.1 g *vs.* 30.0 g±2.1 g; *P>*0.05). The mouse growth rate was consistent with the supplier’s trend description for the strain and age at the end of the experiment. We estimated the parasitic load by counting the recovered trophozoites from the duodenum. Figure 1 shows the *G. lamblia* trophozoite kinetics in male C3H/HeN mice. *Giardia* trophozoites could colonize and stay in the small intestine for 28 dpi. The maximum parasitic recovery in both groups was in the acute infection phase until 6 dpi (*P<*0.05). There was a lower parasite load in the CLA group than in the control group, although the difference was not significant (1×10^4^ ± 0.7×10^4^ *vs.* 3×10^4^±1.2×10^4^ trophozoites/cm, respectively; *P=*0.22). Then, in both groups the trophozoite count decreased progressively from 14 dpi (*P<*0.05) until clearance of infection at 40 dpi (*P>*0.05). 

***sIgA response***

**Figure 2:**
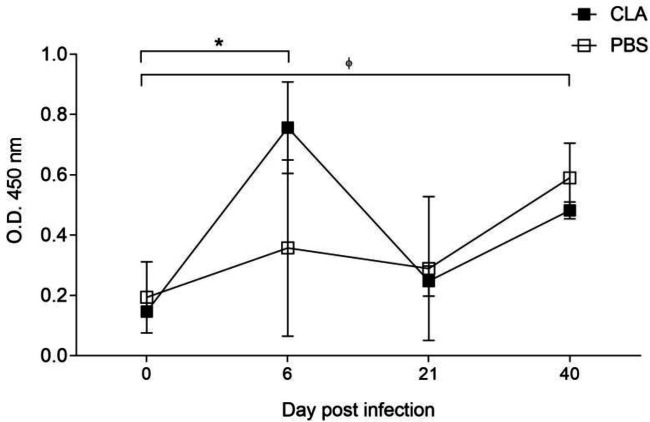
Secretory IgA (sIgA) response of male C3H/HeN mice against *Giardia lamblia*

We assessed mucosal sIgA production weekly in feces before and after infection to evaluate the effect of CLA on the humoral immune response. The infected CLA-supplemented group showed higher *Giardia*-specific sIgA levels than the infected control group at 6 dpi (0.776±0.13 *vs.* 0.357±0.2 optical density). There was a positive humoral response in both infected groups. Still, CLA induced an earlier significant response at 6 dpi (*P=*0.012), while the control group had significant increases in mucosal antibody levels at 40 dpi. Although there were no significant differences between the treatments, CLA could induce greater IgA production because PBS only significantly increased IgA production at 40 dpi compared with the non-infected group (Figure 2). 

***Cytokine production in ex vivo cell challenge***

**Figure 3:**
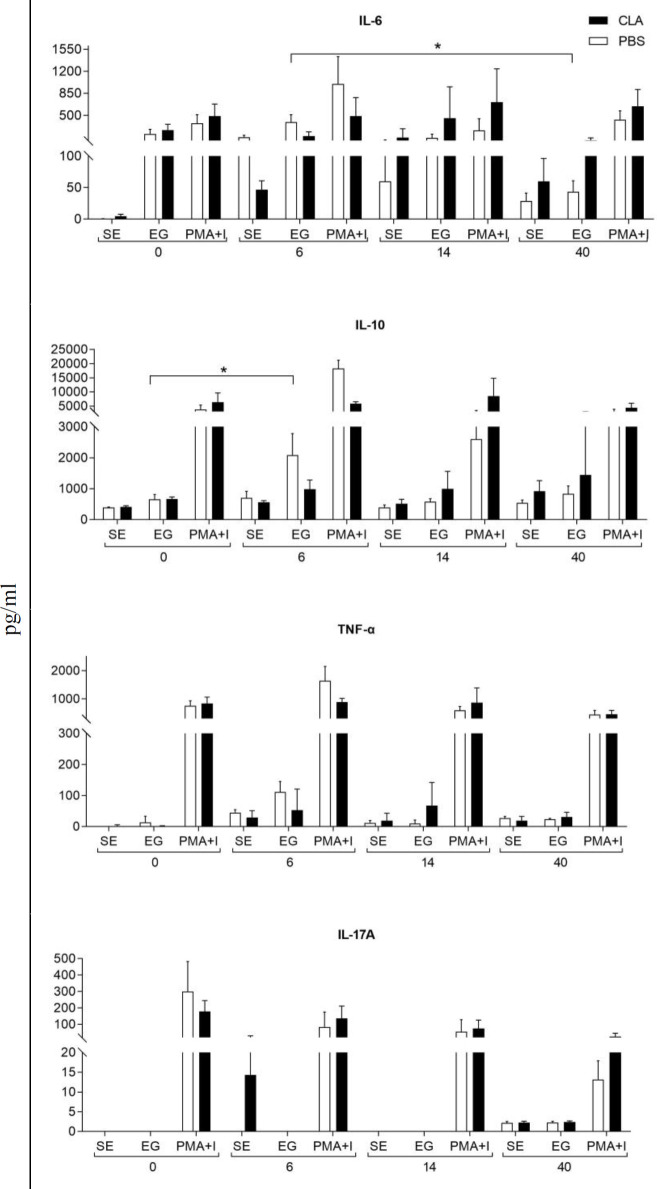
Effect of conjugated linoleic acid (CLA) on cytokine production in *ex vivo* culture supernatant of spleen cells from mice infected with *Giardia lamblia* on days 0, 6, 14, and 40 post-infection

To identify which cytokines were preferably produced against the parasite, we *in vitro*–stimulated spleen cells of the CLA and control groups. We used culture supernatant to quantify cytokine production, including interleukin (IL)-6, IL-10, IL-17A, tumor necrosis factor (TNF)-α, IL-1β, and transforming growth factor (TGF)-β. Only IL-6, IL-10, IL-17A, and TNFα were detectable (Figure 3). IL-6 was produced in spleen cells stimulated with *G. lamblia* extract from the CLA group, but the production was not different compared with spleen cells from the control group. In the control group, IL-6 increased at 6 dpi by *G. lamblia* extract compared with the non-infected group (day 0) but not significantly, although differences were observed at 14 dpi (*P=*0.0103) (Figure 3). In the CLA group, there was a slight increase in IL-6 production in *Giardia* extract–stimulated cells at 14 dpi, albeit not significant. Figure 3 also shows the concentration of IL-10, an anti-inflammatory cytokine. *G. lamblia*–stimulated cells showed elevated IL-10 production at 6 dpi (*P=*0.0456), while TNF-α was also elevated but not significantly (*P=*0.0661) (Figure 3). There were no differences in IL-17A levels for the CLA or control group. 

***Gene expression analysis of nuclear factors and the Treg and Th17 cytokine responses***

**Figure 4:**
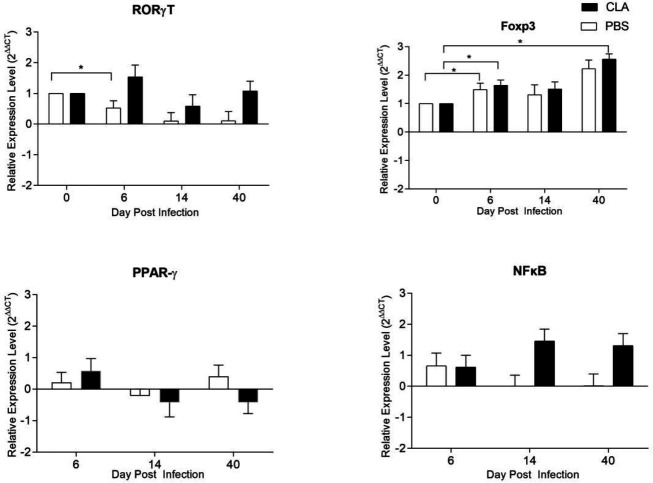
Nuclear factor transcriptional profile of *Giardia lamblia*-infected intestinal cells

The expression of transcription factors and cytokines was measured in intestinal cells by RT-qPCR at 0, 6, 14, and, 40 dpi. Figure 4 shows the expression of the transcription factors RORγT, Foxp3, PPARγ, and NF-κB. RORγT expression was not significantly different between the infected CLA and control groups, but there was a significant difference for the PBS group at 6 dpi compared with 0 dpi (*P=*0.016) (Figure 4). Foxp3 expression was increased at 6 dpi in both groups (*P<*0.05). At 40 dpi in the CLA group, Foxp3 was higher compared with 0 dpi (*P=*0.001) (Figure 4). CLA supplementation or infection evolution had no effect on PPARγ or NF-κB (Figure 4). 

**Figure 5:**
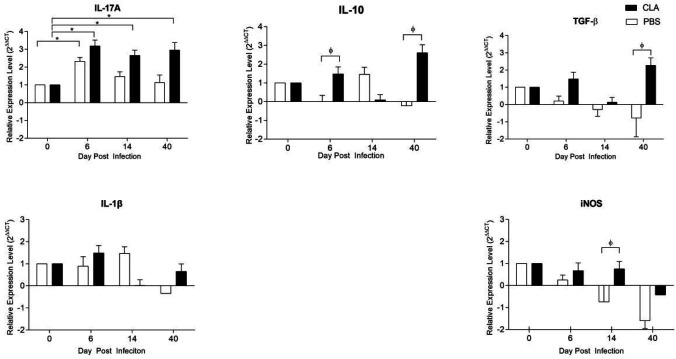
Cytokine transcriptional profile of intestinal cells from *Giardia lamblia*–infected mice

Cytokine production and transcription factor activation are essential for T cell differentiation. CLA supplementation maintained the constant expression of IL-17A at 6, 14 and 40 dpi, while in the control group, IL-17A expression decreased gradually at 14 and 40 dpi, although it was not significantly different from the CLA group (Figure 5). CLA supplementation increased IL-10 expression compared with the control group (*P<*0.05) at 6 and 40 dpi. In contrast, infection had no significant effect on IL-10 production (Figure 5). At 40 dpi, TGF-β expression was significantly higher in the CLA group compared with the control group (*P=*0.047) (Figure 5). *Giardia* infection did not affect IL-1β expression (Figure 5). The expression of inducible nitric oxide synthase (iNOS), which is involved in another branch of antiparasitic responses, was significantly different between the CLA and control groups at 14 dpi (*P=*0.001). It was downregulated at 40 dpi compared with 6 dpi, but the difference was not significant (*P=*0.139). 

***Colon histology***

**Figure 6:**
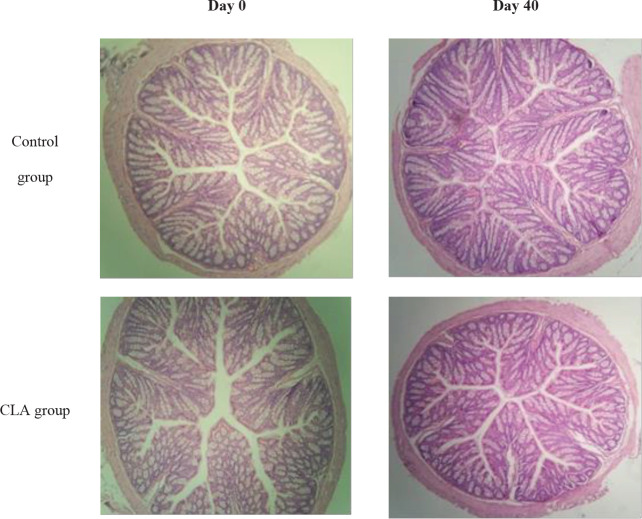
Representative photomicrographs of hematoxylin-eosin-stained colon sections of *Giardia lamblia*–infected mice

We collected samples of the ascending colon at day 0 (pre-infection) and at 40 dpi (post-parasite elimination) to assess changes in the mucosa after the expulsion of *G. lamblia*. The CLA and PBS groups showed no histological damage and intestinal villi presented a typical arrangement without pathological changes in their length (Figure 6).

**Table 1:** Primer sequences used for RT-qPCR amplification of target genes

Sequence	Sense (5′ → 3′)		Antisense (5′ → 3′)		Reference	
RORγT	GAACCAGAACAGGGTCCAGA		CGTAGAAGGTCCTCCAGTCG		(25)	
Foxp3	GGCCCTTCTCCAGGACAGA		GCTGATCATGGCTGGGTTGT		(26)	
PPARγ	TCGCTGATGCACTGCCTATG		GAGAGGTCCACAGAGCTGATT		(27)	
NF-κB	CAGGTCCACTGTCTGCCTCT		TGTCACTATCCCGGAGTTCA		(27)	
IL-17A	ACTACCTCAACCGTTCCACG		TTCCCTCCGCATTGACACAG		(28)	
IL-10	GCTAACCGACTCCTTAATGCAG		AGCTTCTCACCCAGGGAATT		(25)	
TGFβ	TTGCTTCAGCTCCACAGAGA		TGGTTGTAGAGGGCAAGGAC		(25)	
IL-1β	TTCAGGCAGGCAGTATCA		CCAGCAGGTTATCATCATCATC		(29)	
iNOS	CCACGGACGAGACGGATAG		TGGGAGGAGCTGATGGAGTAG		(29)	
β-Actin	CAGCCTTCCTTCTTGGGTAT		TGGGATAGAGGTCTTTACGG		(30)	
			

The intestinal trophozoite count (mean with standard deviation) in C3H/HeN mice before infection (day 0) and after infection (6-40 days post-infection) with *G. lamblia* in the conjugated linoleic acid (CLA)-supplemented and phosphate-buffered saline (PBS)-treated groups. The data represent the mean±standard deviation from individual measurements (n=4 mice per group and per time) in two independent experiments. The asterisk indicates a significant differences (*P<*0.05) between day zero and 6 days post-infection day in both groups

The time course of soluble IgA production was evaluated in feces by enzyme-linked immunosorbent assay. The black squares represent the conjugated linoleic acid (CLA)-supplemented group and the white squares represent the phosphate-buffered saline (PBS)-supplemented group antibody levels before infection (0) and after infection (6-40 days post-infection). The asterisk indicates a significant difference between the CLA infected and non-infected groups (*P=*0.002). ɸ indicates a significant differences between the PBS-supplemented infected and non-infected groups (*P=*0.046). The data represent the mean±standard deviation from individual measurements (n=4 mice per group at each time) in two independent experiments

There are three groups: without stimulation (SE), stimulated with phorbol-myristate-acetate and ionomycin (PMA+I), and with *G. lamblia* extract (EG). The data represent the mean ± standard deviation (n=3 mice per time and per group). Asterisks indicate a significant difference between the time points for the same group (*P<*0.05)

The data represent the mean±standard deviation (n=3 mice per time and per group). Asterisks indicate a significant difference between the time points for the same group (*P<*0.05)

The black bars indicate the conjugated linoleic acid (CLA) group and the white bars represent the phosphate-buffered saline (PBS) group. The data represent the mean±standard deviation (n=3 mice per time-group). ɸ indicates a significant difference between the groups (*P<*0.05); * indicates a significant difference between the time points for the same group (*P<*0.05)

Pre-infection (day 0) and post-elimination stage (day 40) from the control (phosphate-buffered saline) and the conjugated linoleic acid (CLA) groups. The total magnification is 100×

## Discussion

We assessed the effects of CLA oral supplementation on the experimental infection with *G. lamblia* and the induced responses on adaptive cellular and humoral IgA. The parasitic load (Figure 1) peaked at 6 dpi, which is similar to other published *Giardia* infection models (20, 21), and oral CLA administration diminished the *G. lamblia* load at 6 dpi, in the acute phase, and there was elimination at 40 dpi in both infected groups. This is relevant because immunocompetent mice can clear the infection in 3-4 weeks. The humoral immune response is essential to parasitic neutralization and infection clearance (5). The immunomodulatory properties of CLA have been reported in animal and human studies (31), including the effect of CLA on immunoglobulin isotypes IgA, IgG, and IgM (20, 21, 32, 33), also effects in some heamatological parameters (34). In our study, the infected, CLA-treated group showed a twofold increase in* Giardia*-specific sIgA levels at 6 dpi compared with the infected, PBS-treated group (0.776±0.13 vs. 0.357±0.2 optical density). Although there were no significant differences between the treatments, the CLA group showed early IgA production compared with the PBS group: In the latter group, sIgA production was only significantly higher at 40 dpi compared with the non-infected group (Figure 2).

We observed an improvement in IgA production with a short administration period of CLA formulation as free fatty acids. Interestingly, we observed a negative correlation between the specific parasitic load and IgA production (r= -0.307, *P=*0.042). Thus, early mucosal IgA may partly explain the parasite load reduction at 6 dpi in the CLA group (Figure 1). To our knowledge, this study is the first contribution to evaluate the effect of CLA on the IgA mucosal response in *Giardia* parasitic infection. These findings are relevant given the key role that sIgA plays in adaptive immunity against *G. lamblia *(6, 7, 33, 35-37)*.*

Previous reports have shown that CLA might influence important effector T cells, such as Treg and Th17 populations in inductive and effector mucosal sites (38). We assessed IL-17A in the *G. lamblia*–stimulated cells (Figure 3) because this cytokine is essential for transporting high-affinity IgA antibodies required to eliminate *G. lamblia *(28). Recent studies in *Giardia* *muris* and *G. lamblia* models of infection have recognized the crucial role of IL-17, mainly produced by the Th17 lymphocyte population and other mucosal intestinal cells, in parasite immune response and elimination (25, 39). According to Dann *et al.* (28), mice infected with *G. lamblia *reached the peak of infection within the first week, with increased IL-17A expression at 5 dpi that remained high at 14 dpi. However, in a murine model of inflammation, CLA supplementation decreased the expression of pro-inflammatory cytokines, including IL-17A (40). In cells cultured with *G. lamblia* extract, IL-17A was only detectable on 6 dpi in the control group. As reported by Kamda *et al.* (41), IL-6 production by dendritic and other cells is necessary for the induction of Th17 cells that, in turn, produce IL-17A. In mice supplemented with 50:50 CLA, IL-17A was elevated in mesenteric lymph nodes and the spleen in a colitis model (42). On the contrary, Draper *et al.* (40)observed that CLA decreased IL-17A production *in vitro* in mouse cells derived from bone marrow stimulated with ovalbumin peptide. The finding on the maintenance of IL-17A by CLA (Figure 5) could explain, at least in part, the increase in IgA antibodies specific for *G. lamblia* at 6 dpi in the CLA compared with the control group (Figure 2). 

Figure 4 shows the expression the transcription factors RORγT, Foxp3, PPARγ and NF-κB. RORγT is expressed by Th17 cells and has been described as essential in parasite elimination. When comparing our results to those of previously published studies, it must be pointed out that Dreesen *et al.* (25) reported an increase in the transcription profile of RORγT 5 and 6 dpi in C57BL/6 mice infected with *G. muris.* CLA is recognized as a ligand and a potent dual activator of the transcription factors peroxisome proliferator-activated receptor-gamma and alpha (PPARγ and PPARα, respectively) (28, 43). Some CLA effects on immune cells have been explained through PPARγ dependent mechanisms, mainly anti-inflammatory activity by NF-kB suppression (44). However, discrepancies in pro-inflammatory cytokine targets have been found (45). Dreesen *et al.* (25) reported no increase in PPARγ expression in mice infected with *G. muris*. 

In the present assay, the immunological challenge of giardiasis was not strong enough to induce inflammation, which is consistent with the lack of effect of CLA supplementation on whole Treg profile differentiation. In agreement with previous work on mouse models of inflammation, CLA inhibits the expression of NF-κB and pro-inflammatory cytokines (46). Loscher *et al.* (47) reported that CLA decreased IL-12 and increased 1L-10 cytokine production in LPS-stimulated BALB/c mouse cells *in vitro*. PPARγ deactivates NF-κB, but we found no differences in NF-κB in the CLA-supplemented group, contrary to the findings of Borniquel *et al.* (48), who found a decrease of NF-κB in a mouse model of inflammatory bowel disease fed with CLA. 

CLA-supplemented mice shown a significative increase in IL-10 expression compared with the control group (*P≤*0.05). In contrast, IL-10 production was not affected in the control (Figure 5), as previously shown for *G. muris* infection. (25). Reynolds *et al.* (49) found that in mice with lipopolysaccharide (LPS)-induced intestinal inflammation, a diet high in *cis*-9,*trans*-11 CLA increased IL-10 expression at 12 h post-stimulation. Similarly, McCarthy *et al.* (50) described that CLA 80:20 increases IL-10 production at a systemic level. IL-10 has been reported to counteract the production of pro-inflammatory cytokines. Yahya *et al.* (51) observed that in humans infected with *G. lamblia*, there were more Foxp3^+^ Tregs than in the control group. They reported that due to this change, giardiasis induces minimal inflammation in the intestinal mucosa of humans and mice. In our study, the increased Foxp3 and IL-10 expression suggests that Treg proliferation could be favored after infection. Previously, Montalvo-Corral *et al.* (23)showed that CLA supplementation in a C3H/HeN murine giardiasis model affects the CD103^+^ antigen-presenting cell population, which is involved with Tregs. Moreover, the presence of TGF-β has been shown to induce plasticity in Th17 cells by making them IL-10-producing, Treg ex-Th17 cells (52).

The presence of an inflammatory response in giardiasis is controversial. Chen *et al.* (53) observed an increase in IL-1β at 35 dpi in the intestine of mice infected by *G. lamblia*. However, Dreesen *et al.* (25) reported no increase in pro-inflammatory cytokines during the parasite elimination phase. In a model of viral infection with CLA supplementation, Pinelli-Saavedra *et al.* (38) did not find changes in iNOS expression. Still, they observed increased IL-10 production due to the effect of CLA, agreeing with our findings. The colon histology (Figure 6) was similar to that reported by Chen *et al.* (53): They described no changes in mucosal morphology at 35 dpi in mice infected with *G. lamblia*. They also measured occludin, an essential component that provides permeability to epithelial cells, which did not show changes between infected and non-infected mice. In some histological studies of murine models of colitis and humans, authors have found that CLA has a powerful anti-inflammatory effect (42, 54). In mice, the immunological challenge of colitis promotes a severe inflammatory state compared with giardiasis; it is feasible that no significant changes were induced in the colon mucosa of our model.

This study has provided insight into the effects of CLA in the expression of transcription factors and cytokines as well as IgA production that help to downregulate the parasitic load. Those cell populations have an essential role in effector and regulatory responses, and they are important players in B cell plasma differentiation to IgA producers. So, this could be a potential mechanism of CLA action, which may account for the *Giardia* parasitic load reduction. We recommend evaluating whether these findings have clinical or physiological relevance to intestinal health in the post-elimination phase and to define the contribution of the diverse cell populations in producing the cytokines that have been assessed. Our results have revealed a novel anti-parasitic CLA effect mediated by immunomodulatory activity that warrants further study. Therefore, an immunonutritional approach may be helpful to find nutrients with particular effects against parasites and to discover their therapeutic potential.

## Conclusion

CLA induced early production of sIgA related to parasitic load reduction and IL-10 upregulation. Regulatory cytokines and nuclear transcription factors promoting the expression of Tregs contribute to a microenvironment that possibly favors intestinal balance, preventing mucosal damage in the parasite post-elimination stage and disease resolution.

## Authors’ Contributions

MMC and SYMC conceived the study. MMC designed and supervised the experiments; LBC performed IgA ELISA and trophozoite counts experiments; IRD conducted cytokine ELISA and gene expression analysis; GLC carried out histological analysis; IRD, LBC, AJBE, OAB, HPS and MMC analyzed and interpreted the data and prepared figures; SYMC and MMC contributed reagents/materials/analysis tools; MMC, IRD and AJBE wrote the first draft of the manuscript, and all authors (IRD, AJBE, LBC, GLC, OAB, HPS, SYMC, MMC) critically reviewed, contributed, edited and approved the final version of the article. 

## Conflicts of Interest

All authors declare that there are no conflicts of interest.
